# A liquid-crystalline hexagonal columnar phase in highly-dilute suspensions of imogolite nanotubes

**DOI:** 10.1038/ncomms10271

**Published:** 2016-01-05

**Authors:** Erwan Paineau, Marie-Eve M. Krapf, Mohamed-Salah Amara, Natalia V. Matskova, Ivan Dozov, Stéphan Rouzière, Antoine Thill, Pascale Launois, Patrick Davidson

**Affiliations:** 1 Laboratoire de Physique des Solides, CNRS, Univ. Paris-Sud, Université Paris-Saclay, Orsay 91405, France; 2 LIONS, NIMBE, CEA, CNRS, Université Paris-Saclay, CEA-Saclay, Gif sur Yvette 91191, France

## Abstract

Liquid crystals have found wide applications in many fields ranging from detergents to information displays and they are also increasingly being used in the ‘bottom-up' self-assembly approach of material nano-structuration. Moreover, liquid-crystalline organizations are frequently observed by biologists. Here we show that one of the four major lyotropic liquid-crystal phases, the columnar one, is much more stable on dilution than reported so far in literature. Indeed, aqueous suspensions of imogolite nanotubes, at low ionic strength, display the columnar liquid-crystal phase at volume fractions as low as ∼0.2%. Consequently, due to its low visco-elasticity, this columnar phase is easily aligned in an alternating current electric field, in contrast with usual columnar liquid-crystal phases. These findings should have important implications for the statistical physics of the suspensions of charged rods and could also be exploited in materials science to prepare ordered nanocomposites and in biophysics to better understand solutions of rod-like biopolymers.

## Abstract (web summary)

Liquid crystals are grouped into four main classes—nematic, lamellar, cubic and columnar—depending on their symmetries. Here, the authors show for the first time that a columnar phase can form in suspensions of imogolite nanotubes at very low concentrations.

## Graphical abstract

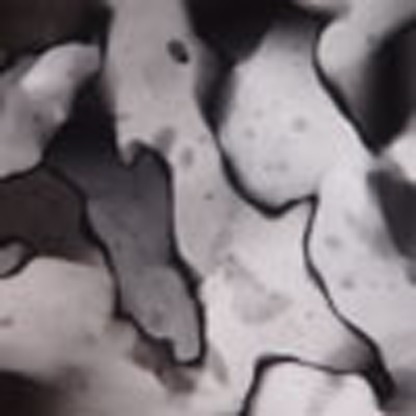

Lyotropic liquid crystals are obtained when rod-like objects (stiff polymers, detergent micelles, nanoparticles) are dispersed in a solvent. According to their symmetries, liquid-crystalline phases belong to four main classes: nematic, lamellar, columnar and cubic^1^. On dilution, the nematic phase remains stable down to very low concentrations (∼0.1%) if its constituents have a large enough aspect ratio^2^. Lamellar and cubic phases stable at low concentrations (∼1%) were also reported^3^,^4^,^5^,^6^. In contrast, the columnar phase, which occurs in suspensions of DNA, rod-like viruses, surfactant micelles and metal oxide nanorods, was only detected at large concentrations (∼10–70%)^7^,^8^,^9^,^10^,^11^.

Imogolite nanotubes (INTs) which were first discovered in soils formed from volcanic ash are the aluminosilicate counterparts of carbon nanotubes (CNT)^12^,^13^. However, unlike the latter, INT are easily synthesized in large amounts by hydrothermal techniques, without need for post-synthesis purification steps, and they are readily dispersed in aqueous solvents to form stable colloidal suspensions^14^,^15^. INT are very similar to CNT in terms of dimensions, aspect ratios and rigidity. They could therefore be used for the same kind of applications as CNT, where geometrical features are important, such as nanostructuring agents or for the elaboration of composite materials. Indeed, both types of nanotubes have comparable Young moduli (2–10 × 10^8^ Pa) but INTs are much easier to functionalize than CNT, which could help optimizing interfacial interactions with the matrix. Moreover, germanium-based (Ge-INT) INTs, similar to usual silicon-based INTs (Si-INT), are easily synthesized from germanium precursors. Furthermore, like CNT, INT occur as single-wall (SW) or double-wall (DW) nanotubes. As expected for highly elongated nanorods, suspensions of INT form a nematic phase at very low volume fractions^16^.

Here we report on the observation of a columnar phase in suspensions of INTs at concentrations around 0.3%. The stability domain of the columnar phase therefore extends to much lower concentrations than previously inferred^17^,^18^,^19^. Moreover, this dilute phase has low visco-elasticity and can easily be aligned in an electric field, unlike other lyotropic columnar mesophases.

## Results

### Nanotube characterization

**Figure 1:**
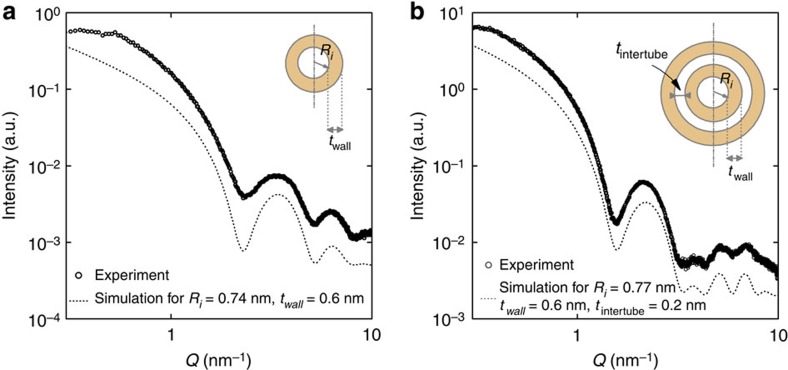
WAXS measurements of imogolite nanotubes. Experimental (open circles) and calculated (dotted line) WAXS curves of (**a**) SW Si-INT and (**b**) DW Ge-INT in suspension. The insets represent sketches of a single-walled and a double-walled nanotube. For the sake of clarity, calculated curves (using equations (1) and (3)) have been translated vertically.

Two different types of INT were investigated: SW Si-INT and DW Ge-INT. Wide-angle X-ray scattering measurement (WAXS) (Fig. 1) was carried out to determine the diameter of the nanotubes. For sufficiently small scattering wave-vectors, that is, <10 nm^−1^, the WAXS diagrams can be fitted within the homogeneous approximation: imogolite SW nanotubes can be modelled by a homogeneous hollow cylinder with an internal radius *R*_i_, a wall thickness *t*_wall_ and an average electronic density *ρ*_INT_. The external radius is *R*_e_=*R*_i_+*t*_wall_ (inset Fig. 1a). DW nanotubes can be modelled by two concentric cylinders with the same wall thickness. The external radius of DW nanotubes is thus *R*_e_=*R*_i_+2*t*_wall_+*t*_intertube_ with *t*_intertube_ the gap between the inner and outer tubes (inset Fig. 1b).

The scattered X-ray intensity for individual SW nanotubes well-dispersed in suspension writes:


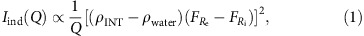


where *F*_*R*_ refers to the Fourier transform of the projection of a full cylinder of radius *R* along its axis:


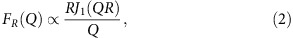


with *J*_1_ the cylindrical Bessel function of order 1; *ρ*_water_ is the water electronic density inside and outside the cylinder, assumed to be the same in first approximation. For individual DW nanotubes in suspension, one finds:


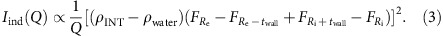


Equations (1) and (3) were used to fit the experimental scattering curves in Fig. 1 and to estimate nanotube diameters. SW Si-INT and DW Ge-INT have, respectively, diameters of 2.7 nm and 4.3 nm and lengths of about 700 nm and 500 nm, the latter being determined by atomic force microscopy (AFM) (Supplementary Fig. 1). The length of the nanotubes is very polydisperse (∼55% and 77%, respectively) but their diameter is well-defined (∼5%)^15^.

### Polarized optical microscopy

**Figure 2:**
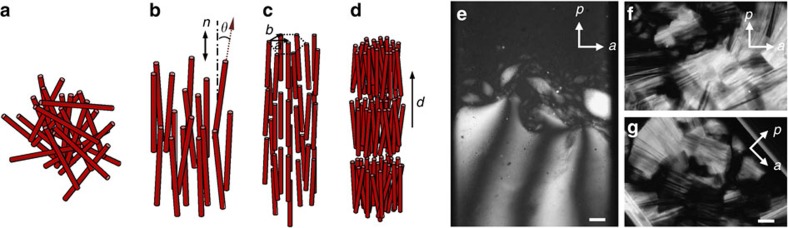
Liquid-crystal phases in imogolite suspensions. Schematic representations of (**a**) isotropic liquid, (**b**) nematic, (**c**) columnar and (**d**) smectic liquid-crystal phases. Texture photographs of (**e**) isotropic/nematic phase coexistence with sedimenting spindle-shaped nematic droplets (*φ*=0.07%) and (**f**,**g**) columnar phase (*φ*=0.31%) obtained with SW Si-INT suspensions. Scale bar, 200 μm.

**Figure 3:**
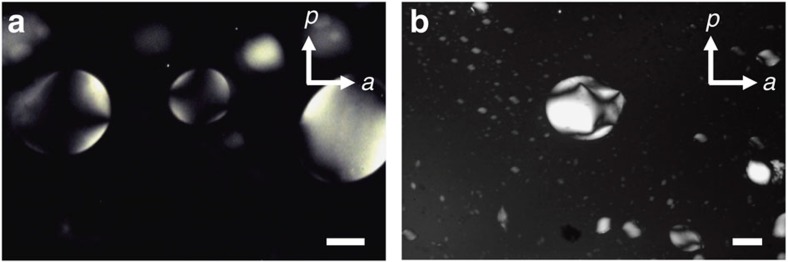
Nematic tactoids in imogolite suspensions. Texture photographs of (**a**) SW Si-INT (*φ*=0.07%) and (**b**) DW Ge-INT (*φ*=0.12%) suspensions. Scale bar, 200 μm.

In polarized-light microscopy, the suspensions of INTs exhibit a clear phase separation at very low volume fractions *φ* (Fig. 2). The bottom phase is birefringent and denser than the isotropic (I) top phase, which is not devoid of INT because it shows strong flow-birefringence. The schlieren texture of the bottom phase proves that it is nematic (N). Indeed, in freshly prepared samples, nematic droplets, called tactoids, form in a few days and sediment, both in test-tubes and in capillaries stored vertically (Fig. 3). This first order I/N transition, already reported for other suspensions of INT, is well-described by the Onsager model^2^,^15^,^16^,^20^. However, at slightly larger volume fractions (Fig. 2b,c), very different textures suggest the existence of another liquid-crystalline phase but do not allow unambiguous identification of its nature.

### Small-angle X-ray scattering experiments

**Figure 4:**
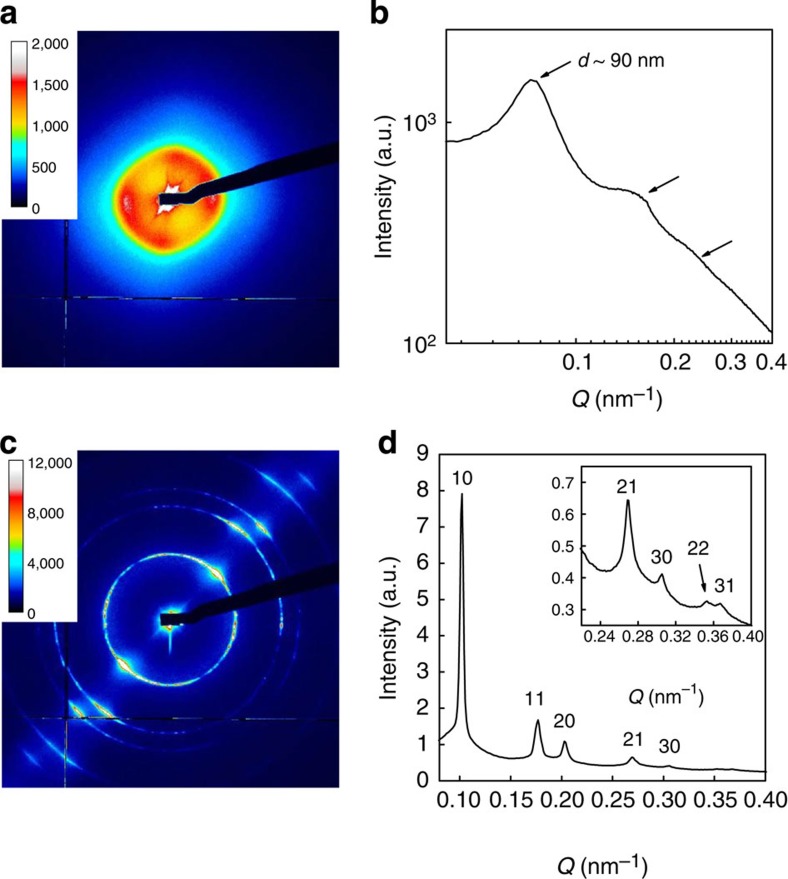
SAXS measurements of DW Ge-INT suspensions. SAXS patterns of (**a**) nematic (*φ*=0.12%) and (**c**) columnar (*φ*=0.22%) DW Ge-INT suspensions and (**b**,**d**) their corresponding *I*(*Q*) curves. (The hk indices in **d** refer to the 2D hexagonal reciprocal lattice peaks.)

**Figure 5:**
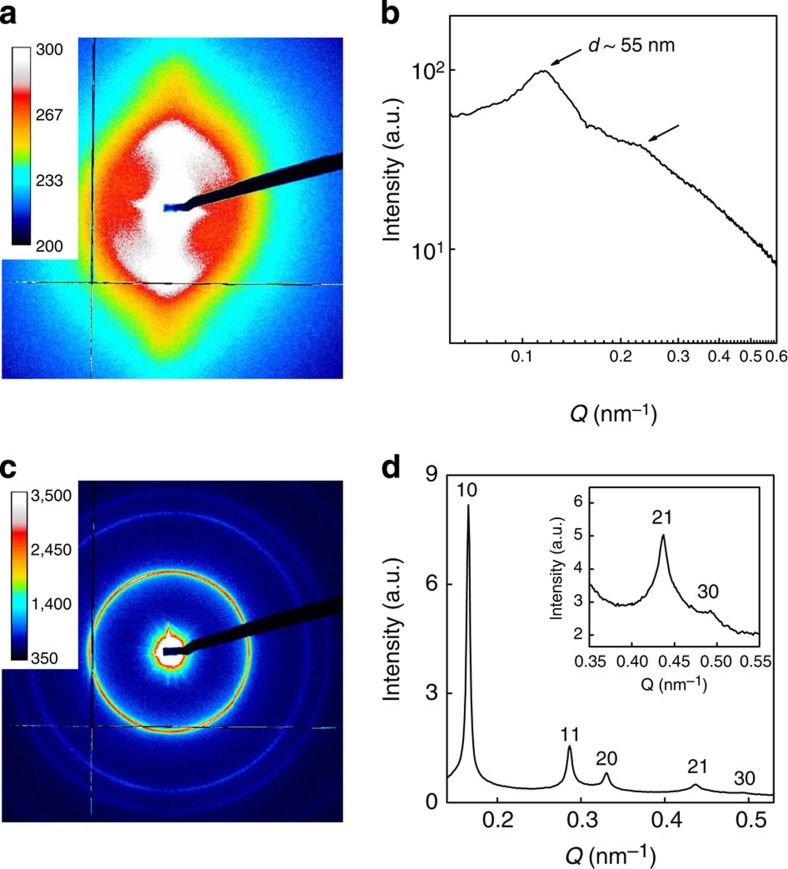
SAXS measurements of SW Si-INT suspensions. SAXS patterns of (**a**) nematic (*φ*=0.14%) and (**c**) columnar (*φ*=0.31%) SW Si-INT suspensions and (**b**,**d**) their corresponding *I*(*Q*) curves. (The hk indices in **d** refer to the 2D hexagonal reciprocal lattice peaks.)

The small-angle X-ray scattering (SAXS) patterns of the nematic phase (Figs 4a,b and 5a,b) do not show any sharp reflection, as expected. They display several broad scattering rings (arrows in Figs 4b and 5b) whose positions depend on volume fraction. These rings are due to a liquid-like structure factor arising from the local positional order of the INT in the plane perpendicular to their main axis.

In stark contrast, the scattering patterns of the second mesophase display many thin diffraction rings that can all be indexed with a two-dimensional (2D) hexagonal lattice (Fig. 4c,d and Supplementary Fig. 2). Therefore, the phase is of the columnar hexagonal (Col_H_) type where the rods organize on a hexagonal lattice perpendicular to the INT average direction (C_6_ axis). The dilute columnar phase was discovered in suspensions of both SW Si-INT (Fig. 5c,d) and DW Ge-INT, which shows that its occurrence is not related to either nanotube composition or wall structure. Altogether, on increasing volume fraction *φ*, the phase sequence of SW Si-INT is I 0.06% N 0.24% Col_H_ and that of DW Ge-INT is I 0.08% N 0.13% Col_H_. At larger *φ* (∼0.7%), a sol/gel transition occurs that prevents samples from reaching full thermodynamic equilibrium. No lamellar mesophase was observed, most probably because of the large length polydispersity of the INT.

The sharpness of the X-ray reflections allows us to measure accurately the lattice parameter, *a* (Supplementary Fig. 2). Assuming a homogeneous hexagonal columnar phase of nanotubes, one can calculate its volume fraction using the formula: *φ*=(2*π*/3^1/2^)(*R*/*a*)^2^ where *R* is the nanotube radius. For example, for SW Si-INT, we obtain *φ*=0.28% to be compared with the volume fraction of 0.31% measured by slow drying. Similarly, for DW Ge-INT, we obtain 0.25% to be compared with 0.23%. This confirms both the high dilution of the columnar phase and its homogeneity.

Due to the high dilution, the lattice parameter *a* of the columnar phase is quite large; for example, it is 71.3 nm for DW Ge-INT at *φ*=0.22%. This value is more than 15 times larger than the nanorod outer diameter (4.3 nm). Moreover, numerical simulations of the scattering patterns from INT on a 2D hexagonal lattice allowed us to determine the columnar domain size from the widths of the diffraction lines (see Methods section). The typical domain size obtained,∼1.6 μm, in the plane perpendicular to the C_6_ axis, is about 20 times the lattice spacing in a domain, meaning that some 450 INT diffract X-rays coherently in that plane. Correlatively, the observation of 7 orders of diffraction shows that the positional fluctuations of the INT are very low, only a few % of the lattice spacing.

### Alignment under electric field

**Figure 6:**
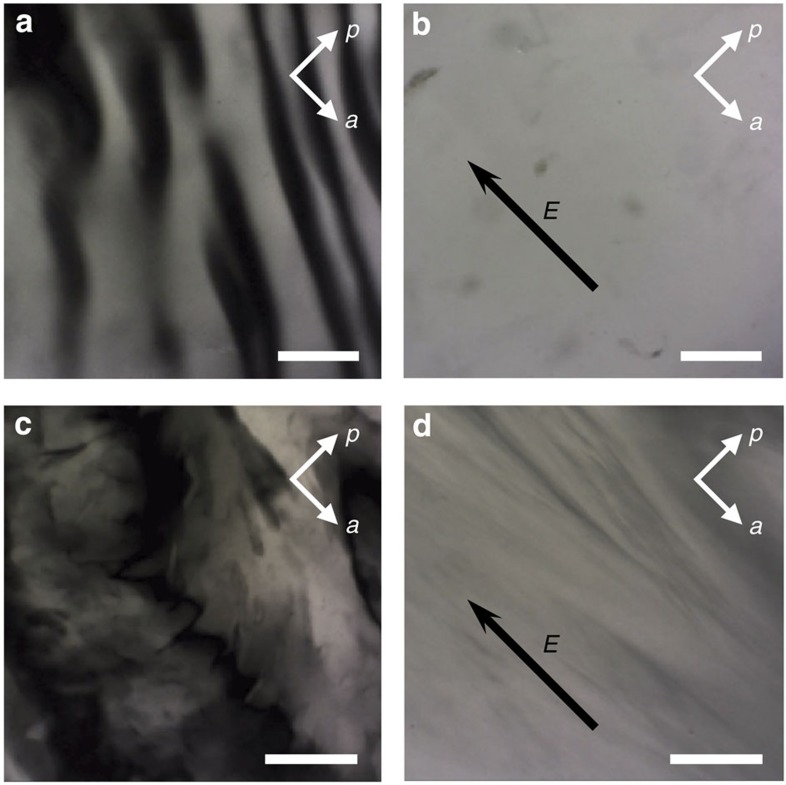
Texture photographs of aligned imogolite suspensions. (**a**,**b**) nematic (*φ*=0.12%) and (**c**,**d**) columnar (*φ*=0.22%) DW Ge-INT suspensions before (**a**,**c**) and after (**b**,**d**) application of an electric field *E*=(100 V mm^−1^, 500 kHz). Scale bar, 100 μm.

INTs in aqueous suspensions readily respond to a high-frequency alternating current electric field because they are highly charged anisotropic objects surrounded by ionic clouds. Indeed, both nematic and columnar phases are easily aligned by applying a moderate field (∼100 V mm^−1^) for about 10 s only (Fig. 6).

**Figure 7:**
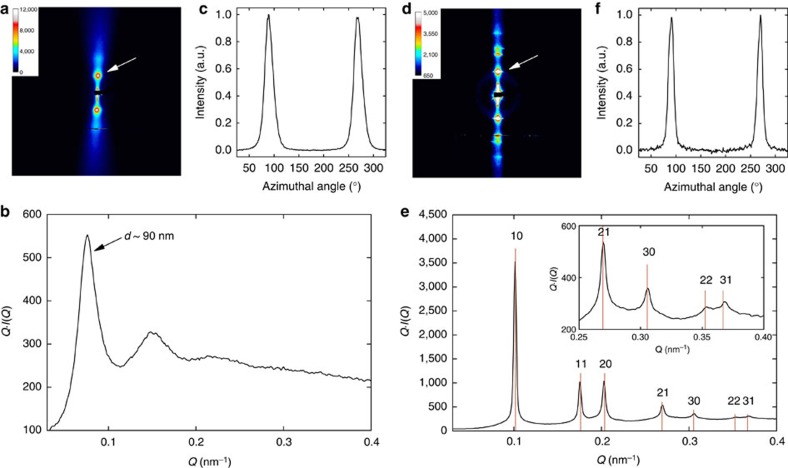
SAXS measurements of aligned imogolite nanotube suspensions. (**a**–**c**) nematic (*φ*=0.12%) and (**d**–**f**) columnar (*φ*=0.22%) DW Ge-INT suspensions submitted to an electric field *E*=100 V mm^−1^ at 500 kHz. (**a**,**d**) 2D-SAXS patterns, (**b**,**e**) structure factor *Q I*(*Q*) and (**c**,**f**) azimuthal profiles recorded along a circle going through the diffuse scattering maximum (white arrow in **a** and **d**). In **e**, the hk indices refer to the 2D hexagonal reciprocal lattice peaks (red lines) of Ge-INT in the columnar phase with lattice parameter *a*=71.3 nm.

**Figure 8:**
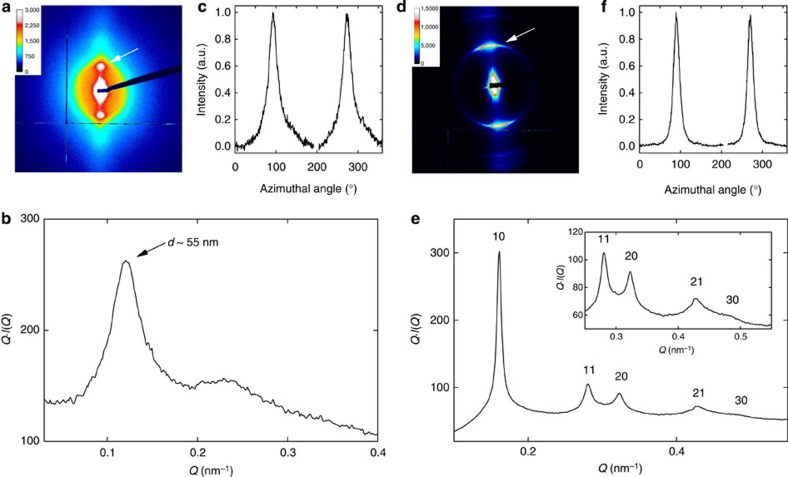
SAXS measurements of aligned Si-INT suspensions. (**a**–**c**) Nematic (*φ*=0.14%) and (**d**-**f**) columnar (*φ*=0.31%) SW Si-INT suspensions submitted to an electric field *E*=200 V mm^−1^ at 500 kHz. (**a**,**d**) 2D-SAXS patterns, (**b**,**e**) structure factor *Q*.*I*(*Q*) and (**c**,**f**) azimuthal profiles recorded along a circle going through the scattering maximum (white arrow in **a** and **d**). In **e**, the hk indices refer to the 2D hexagonal reciprocal lattice peaks.

The birefringence Δ*n* of these columnar monodomains was measured (Δ*n*=8.2 × 10^−5^ for SW Si-INT and 1.7 × 10^−4^ for DW Ge-INT), which allows cross-checking the low value of the volume fraction (see Methods section). Scattering patterns of well-aligned INT were recorded from suspensions submitted to an electric field *in situ*, both in the nematic and columnar phases (Figs 7 and 8). These patterns prove that the INT align parallel to the field. The nematic order parameter, *S*=0.90±0.05, is very large, which agrees with the strongly first order character of the I/N transition. In the columnar phase, the dependence of scattered intensity versus azimuthal angle should rather be fitted by a gaussian distribution of columnar crystallites. The small width of this distribution (∼12° full width at half maximum) illustrates the good alignment of the columnar phase in the field, which is very original for such colloidal phases that are usually rather stiff.

## Discussion

The occurrence at large volume fractions of a columnar mesophase is firmly predicted for suspensions of rigid hard rods of polydispersity in length larger than 18% (ref. 18). However, the calculation of the phase diagrams of charged nanorods in polar solvents is still an active research field, due to the intrinsic complexity of the electrostatic interactions in these systems^19^,^21^,^22^. The influence of particle polydispersity is probably less important in these dilute systems than in dense ones but it still needs to be investigated theoretically. Experimentally, the columnar phases of rod-like particles (DNA, viruses, polypeptides, polysaccharides, metallic or mineral nanoparticles...) reported so far occur at volume fractions of ∼10–50% and the lattice spacing is close to the rod diameter. In comparison, the columnar phase of INT occurs at volume fractions that are 2 orders of magnitude lower. Such difference of behaviour is probably related to the huge aspect ratio (100–300) of INTs, which also favours the nematic phase^16^. Moreover, the ionic strength of INT suspensions was kept low on purpose (∼10^−4^ M) to ensure a large Debye length (*κ*^−1^∼30 nm) extending the range of electrostatic repulsions between the charged INT. Indeed, adding salt to the suspensions brings about their flocculation. To account for the very low volume fraction of the columnar phase of charged INT, it is tempting to use a simple renormalization of the phase diagram of hard rods by introducing an effective diameter, *D*_eff_=*D*+2*κ*^−1^. Starting from a typical volume fraction of 0.5 for the onset of the columnar phase of hard rods^18^, with *κ*^−1^=30 nm at an ionic strength of 10^−4^ M, volume fractions of 0.093% and 0.22% are, respectively, obtained for SW Si-INT and DW Ge-INT. These predicted values are in fair agreement with the experimental ones (0.24% and 0.13%, respectively). However, this renormalization procedure is quite dubious because the interaction potential of charged particles is always anisotropic, even at long distance and intrinsically much more complex than that of hard particles, as recently demonstrated in the case of disks^22^. The absence of strong enough electrostatic repulsions in most suspensions of CNT, which could balance the van der Waals attractions, is probably the reason why a similar dilute columnar phase was not yet reported in these systems^23^.

The very low concentration of the columnar phase of INT makes this phase reminiscent of the highly ‘swollen' lamellar phase of surfactant membranes and of the nematic phase of ‘swelling clays'^3^,^24^,^25^. However, the case of nanotubes is even more surprising because they are 1D objects that can fluctuate more easily in orientation and position without colliding than 2D objects like membranes and nanosheets. Moreover, the orientational order in this system is somewhat comparable to that of gold nanorods diluted in a liquid crystal although the interactions in that case are mediated by the matrix^26^,^27^. Furthermore, the 2D positional long-range order of the nanotubes in the columnar phase reminds of the 3D crystalline order of both charged spherical particles in highly dilute colloidal crystals^28^ and charged nanorods in cubic plastic crystals^29^. However, the columnar liquid-crystalline phase is more intricate than the two other systems because of the onset of long-range orientational order.

The remarkable positional ordering of the charged INT is strong evidence of the large intensity of the electrostatic repulsions between charged linear objects, which stabilize the columnar phase down to very low volume fractions. Therefore, the phase diagrams of suspensions of rod-like objects in polar solvents clearly deserve more thorough theoretical investigations. From an applied point of view, the appearance of the columnar phase at high dilution and its easy alignment in an electric field could be the first step towards the elaboration of anisotropic nanocomposite materials with well-dispersed nanotube filler. Indeed, such hybrid materials keeping liquid-crystalline organization can easily be produced by polycondensation of silica precursors under field, as illustrated in the case of chitin nanorods^30^. Moreover, this system that is optically transparent in a wide spectrum and that can be manipulated by moderate electric fields is somewhat similar to a strongly anisotropic 2D photonic crystal. Although the optical contrast is presently too low to really expect photonic bandgap and other spectacular optical properties, it could be enhanced by doping the nanotubes with dyes^31^. This will result in a periodic anisotropic 2D structure with a strong spatial variation of optical properties, such as dichroism and birefringence, with potential photonic applications.

## Methods

### Synthesis of aluminosilicate Si-INT nanotubes

Aluminosilicate nanotubes were synthesized by adding tetraethoxysilane to a dilute (*C*=10^−3^ mol l^−1^) aluminium perchlorate solution in a Teflon beaker. Then, the mixture was slowly hydrolysed by the addition of a 0.1 mol l^−1^ NaOH solution to reach a hydrolysis ratio ([OH]/[Al]) of 2. Solutions were stirred overnight at room temperature and then aged for 5 days into an oven at 90 °C.

### Synthesis of aluminogermanate Ge-INT nanotubes

We recently described the simple single-step synthesis of micron-long germanium imogolite-like nanotubes^15^. Briefly, tetraethoxygermanium was mixed in a Teflon beaker with a 0.2 mol l^−1^ aqueous solution of aluminium perchlorate using the imogolite stoichiometric ratio [Al]/[Ge]=2. Then, a urea (CO(NH_2_)_2_) solution was added at room temperature up to a [urea]/[Al] ratio of 1. Indeed, the thermal decomposition of urea produces *in situ* hydroxyl ions (two hydroxyls per urea molecule) homogeneously distributed in the solution. Immediately after mixing, the solution was transferred in an autoclave and placed in an oven at 140 °C for 5 days.

### Sample preparation

At the end of the aging process, the Si- and Ge-INT mixtures were cooled down and dialysed against ultrapure water using 10-kDa membranes (Visking) until the conductivity fell below 5 μS cm^−1^. Si-INT suspensions, due to their low concentration of aluminium precursor, were concentrated with an ultrafiltration set-up. In both cases (Si and Ge-INT), the pH of the suspensions was close to 6. Samples with different concentrations were then prepared by dilution with ultrapure water and stored in air-tight vials before measurements. For all samples, the mass concentration was determined by weight loss on drying.

### Atomic force microscopy

The average length of Si and Ge-INT samples were measured using AFM (Digital Instruments, Nanoscope V). The tapping mode was employed for imaging nanotubes. Freshly cleaved mica sheets were dipped into very dilute suspensions (∼20 mg l^−1^) for 1 min followed by three cleanings in ultrapure water to remove unabsorbed nanotubes. The mica sheets were then dried at 60 °C overnight before performing measurements.

### Electrostatic parameters of INTs

The point of zero charge (PZC) of synthetic INTs was either taken from literature for SW Si-INT (PZC∼9.5)^32^ or measured by electrophoretic mobility for DW Ge-INT (PZC=11). This means that INTs are positively charged at pH 6. Their ζ-potential, deduced from the measurements of electrophoretic mobility, is +45 mV for SW Si-INT and +50 mV for DW Ge-INT, at this pH. A bare linear charge density of∼0.7e^−^ per nm was estimated for INTs from the measurements of electrophoretic mobility and the nanotube dimensions^33^.

### Polarized-light microscopy

The samples were transferred into thin flat optical glass capillaries (VitroCom, 0.2 × 2 mm), which were flame sealed to avoid water evaporation and stored vertically to follow the phase separation. Optical textures were recorded with a polarizing microscope (BX51-P, Olympus) equipped with a CCD camera.

### Birefringence measurements

The birefringence of the samples was measured directly under the microscope using an Olympus Berek optical compensator. Moreover, using the formula: Δ*n*=Δ*n*_p_*φS*, with *S*∼0.9 and where Δ*n*_p_ is the estimated specific birefringence of the particle (Δ*n*_p_=0.019 for SW Si-INT and 0.063 for DW Ge-INT), the volume fraction of the columnar phase can be retrieved and compares very well with those determined by weight loss on drying, all the more when one considers the approximations involved in the calculation of Δ*n*_p_. For example, we obtain *φ*=0.42% instead of 0.31% for SW Si-INT and *φ*=0.27% instead of 0.23% for DW Ge-INT.

### Electric field set-up

The electric field is applied to the samples along the capillary axis, perpendicular to the X-ray beam or to the direction of optical observation. This geometry presents an important advantage for the *in situ* application of the field in the SAXS experiments, as the electrodes are outside of the observation window and only the thin 10-μm glass walls of the capillary are in the beam path. The electric field set-up is described in details in ref. 34. Briefly, a pair of external electrodes made of aluminium foil, encircles completely the capillary, 2 mm apart along the capillary axis. The electrodes are pressed onto the capillary protected by a cushion of soft foam to optimize the electrode/glass wall contact without damaging the capillary tube. High-frequency (500 kHz) sinusoidal alternating current voltage, either continuous or pulsed, was applied to the electrodes, with amplitudes varying from 0 to 1,100 V. The electric field penetrating into the suspension is moderately strong, up to about 200 V mm^−1^ root mean squared (r.m.s.) value (corrected for the attenuation due to the screening by the mobile charges and the dielectric mismatch between suspension and capillary). The field is parallel to the capillary axis, with highly uniform strength and orientation in the whole inter-electrode area accessible for observation. This electric-field holder was fitted onto either the stage of the polarizing microscope for electro-optical measurements or the experimental table of the SWING beamline at synchrotron SOLEIL, with the capillary tubes (and the electric field) held horizontal for electro-SAXS experiments. We report the experimental results as a function of the applied voltage. The r.m.s. value of the electric field *E* inside the sample was estimated from numerical simulations and frequency-dependent electric-birefringence experiments. Under the present experimental conditions, we obtained *E*=180 × *U*_r.m.s._ (V m^−1^), giving *E*=200 V mm^−1^ for the highest applied voltage, *U*_r.m.s._=1,100 V.

### Small and wide-angle X-ray scattering

SAXS experiments were carried out at the SWING beamline of the synchrotron SOLEIL (Orsay, France). Measurements were performed at a wavelength of *λ*=0.138 nm with an incident X-ray beam of ∼350 × 100 μm^2^ in the horizontal and vertical directions, respectively. 2D scattering patterns were acquired on an AVIEX CCD detector formed by four detectors with 167 μm pixel size and placed in a vacuum detection tunnel, at a sample to detector distance of 6.57 m. The typical accessible range of scattering vector modulus *Q* was 0.01−0.5 nm^−1^ (*Q*=4*π*sin*θ*/*λ*, where 2*θ* is the scattering angle).

Samples of suspensions were filled by mild centrifugation into borosilicate capillary tubes (GLAS, Schönwalde bei Berlin, Germany) of 1 mm diameter and stored vertically after flame-sealing. (Other samples were prepared in 1.5 mm diameter glass tubes and in Kapton tubes as well and showed exactly the same results.)

The sample size in all directions is at least 1,000 times larger than any particle dimension (or spacing). Such ratio rules out any confinement effect^35^ in the appearance of the columnar phase, which must then be of thermodynamic bulk origin. SAXS patterns, in the absence of field, are most often anisotropic because of the spurious partial alignment of the phase and/or the presence of several large domains in the volume illuminated by the X-ray beam.

WAXS measurements were performed using a rotating anode generator (*λ*_MoKα_=0.0711, nm, Rigaku), a multilayer W/Si mirror (Osmic) and home-made hybrid slits, providing a monochromatic beam of 1 × 1 mm^2^ at the sample position. A vacuum chamber behind the sample allows minimization of the X-ray scattering signal of air. The transmitted flux was measured continuously with a photodiode placed in the beam stop. 2D scattering patterns were collected on a MAR research X-ray-sensitive 345-mm plate detector with 100 μm pixel size, placed behind the output window of the vacuum chamber, at a distance of 720 mm from the sample. The typical accessible range of scattering vector modulus *Q* was 0.3−30 nm^−1^. Samples of suspensions were transferred into kapton cylindrical capillaries of 2.6 mm diameter.

In both cases, the curves of scattering intensity versus *Q* were deduced from angular integration over [0, 2*π*] of the X-ray scattering patterns previously corrected for water and glass or kapton scattering. For anisotropic patterns, the angular integration was reduced around the direction of maximum intensity. Furthermore, in the case of samples aligned under electric field, angular profiles of scattered intensity at constant *Q* were also performed. The nematic order parameter, *S*, of the nematic samples was retrieved from a fit of the scattered intensity versus azimuthal angle^36^.

### SAXS diagram of INT organized on a 2D hexagonal lattice

Evaluation of the coherence domain size in the columnar phase was made by comparing the measured peak widths with those obtained by calculating the intensity *I*(*Q*) scattered by domains of columnar phase of extension *D*_domain_, perpendicular to the long axes of INT. One easily obtains^37^:


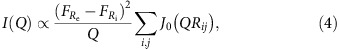


for SW-INT and


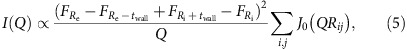


for DW-INT, where *J*_0_ is the cylindrical Bessel function of order zero and the sum runs over all nanotubes *i* and *j* within the columnar domain, *R*_*ij*_ is the distance between nanotubes *i* and *j* perpendicularly to their axis (*R*_*ij*_≤*D*_domain_). Calculated peak widths decrease with increasing domain extension. Small measured peak widths thus lead to large domain sizes.

## Additional information

**How to cite this article:** Paineau, E. *et al.* A liquid-crystalline hexagonal columnar phase in highly-dilute suspensions of imogolite nanotubes. *Nat. Commun.* 7:10271 doi: 10.1038/ncomms10271 (2016).

## Supplementary Material

Supplementary Information Supplementary Figures 1-2
