# Supplementary material for: A liquid-crystalline hexagonal columnar phase in highly-dilute suspensions of imogolite nanotubes
Source: Nat Commun. 2016 Jan 5;7:10271. doi: 10.1038/ncomms10271 (PMC4728447; doi:10.1038/ncomms10271)
Supplement: Supplementary Information — Supplementary Figures 1-2 [file ncomms10271-s1.pdf]

Supplementary figures

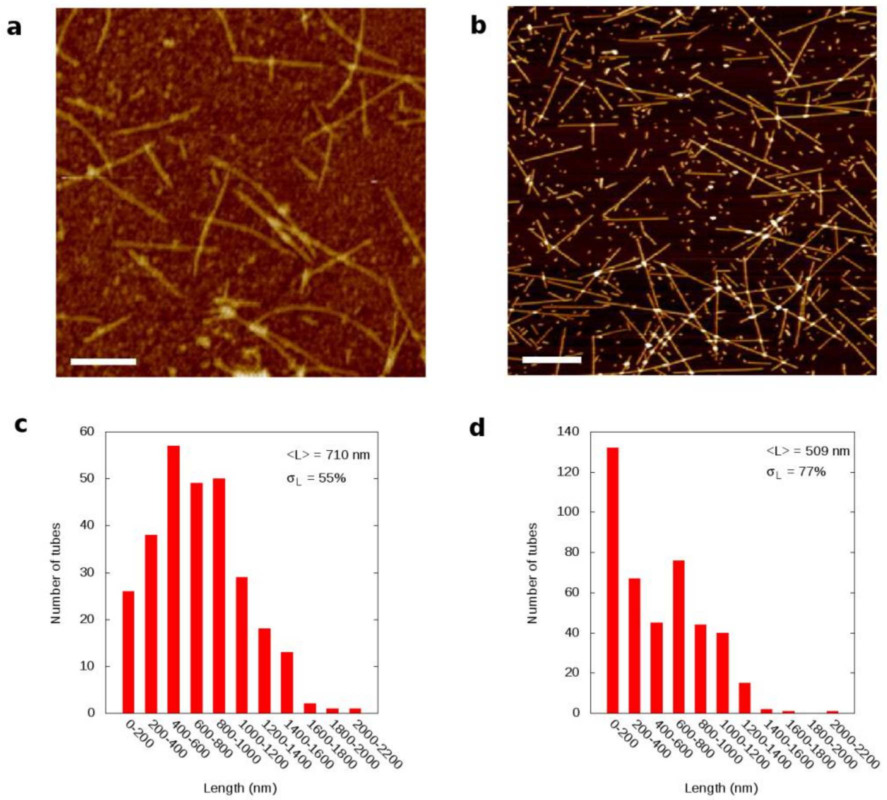

Supplementary Figure 1. Length characterization of imogolite nanotubes. AFM images (top panels) and length distribution histograms (bottom panels) of (a,c) SW Si-INT and (b,d) DW Ge-INT. The scale bar represents 500 nm. <*L*> and σ refer to the average length and the relative standard deviation, respectively.

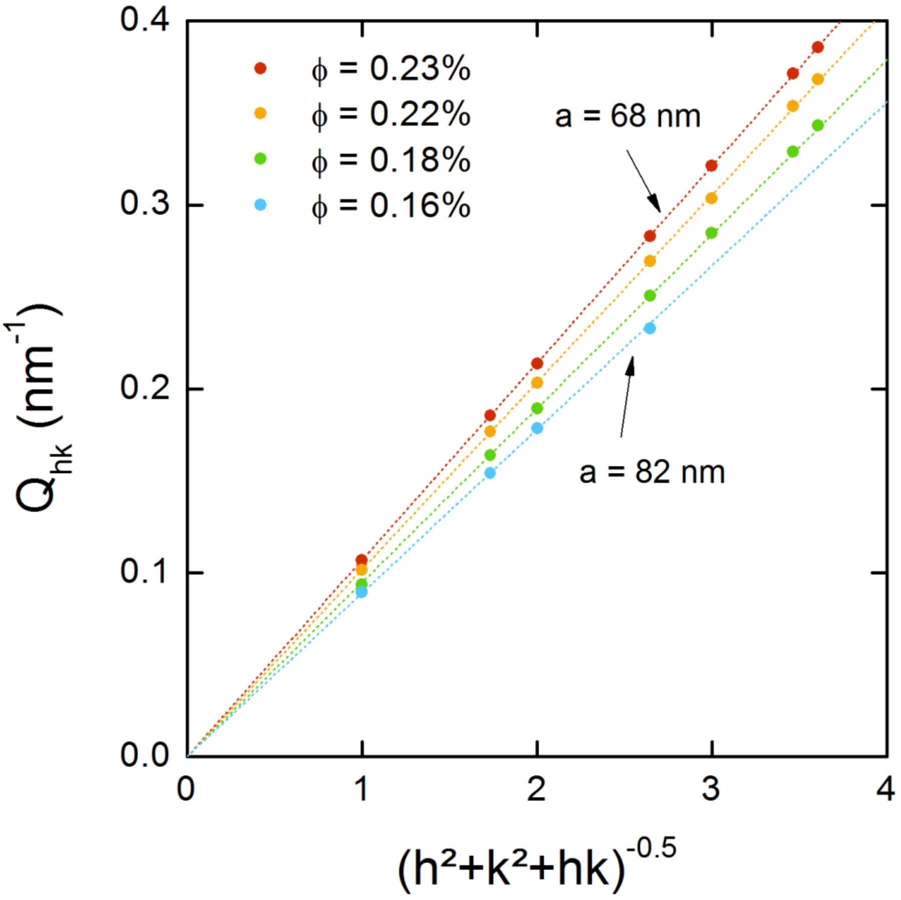

Supplementary Figure 2. Indexation of the SAXS reflections with a hexagonal lattice.

Plots of the *Q* vectors of the hk reflections of DW Ge-INT columnar phases at different volume fractions vs. $(h^2+k^2+hk)^{-0.5}$. The straight lines illustrate the good agreement of the peak positions with the 2D hexagonal lattice.
